# The trade-offs of emotional reactivity for youths' social information processing in the context of maternal depression

**DOI:** 10.3389/fnint.2012.00043

**Published:** 2012-07-13

**Authors:** Megan Flynn, Karen D. Rudolph

**Affiliations:** ^1^Bethel University, St. PaulMN, USA; ^2^University of Illinois, Urbana-Champaign, ChampaignIL, USA

**Keywords:** differential susceptibility, emotional reactivity, maternal psychopathology, social information processing, cognitive bias

## Abstract

Although research demonstrates that emotional experiences can influence cognitive processing, little is known about individual differences in this association, particularly in youth. The present study examined how the emotional backdrop of the caregiving environment, as reflected in exposure to maternal depression and anxiety, was linked to biases in youths' cognitive processing of mother-referent information. Further, we investigated whether this association differed according to variation in youths' emotional reactivity to stress. Youth (50 boys, 46 girls; *M* age = 12.36, SD = 1.05) completed a behavioral task assessing cognitive bias. Semi-structured interviews were administered to assess (a) youths' emotional reactivity to naturally occurring stressors, and (b) maternal depression and anxiety. Hierarchical multiple regression analyses revealed that emotional reactivity to interpersonal stressors moderated the linkage between maternal depression and cognitive bias such that maternal depression predicted a greater negative bias in youth exhibiting high and average, but not low, levels of emotional reactivity. At low levels of maternal depression, youth with heightened interpersonal emotional reactivity showed a greater positive cognitive bias. This pattern of effects was specific to interpersonal (but not non-interpersonal) emotional reactivity and to maternal depression (but not anxiety). These findings illuminate one personal characteristic of youth that moderates emotion-cognition linkages, and reveal that emotional reactivity both enhances and impairs youths' cognitive processing as a function of socialization context.

## Introduction

Despite recognition of mutual and dynamic connections between emotion and cognition at both proximal and distal levels, developmental scientists traditionally have examined normative and atypical emotional and cognitive processes in isolation (Calkins and Bell, 2010). Contemporary experimental paradigms have helped address this gap by examining a variety of integrated emotion-cognition associations, as well as individual differences in these links (e.g., Henderson et al., 2012; for a review, see Dolcos et al., 2011). In particular, cognitive and affective neuroscience research highlights the interplay between characteristics of affect (e.g., emotion generation and regulation) and cognition (e.g., attention, memory). This research suggests that emotional systems can both enhance and impair cognitive functions (i.e., improving versus reducing accuracy and efficiency; for reviews, see De Raedt et al., 2010; Dolcos et al., 2011). Building on prior research and extending it to an interpersonal context, the primary goal of this study was to examine the association between emotional facets of caregiving and youths' cognitive processing, and to explore personal characteristics of youth that determine the strength of this link. In particular, we investigated (a) how the emotional backdrop of the caregiving environment, as reflected in exposure to maternal psychopathology, was linked to biases in youths' cognitive processing of mother-relevant information, and (b) whether the magnitude of this association differed according to variation in youths' emotional reactivity to stress.

### Person X environment models of development

Several theoretical models have been proposed to explain individual differences in sensitivity to environmental experiences. Traditional vulnerability or dual-risk models hold that adverse environmental experiences more strongly predict detrimental outcomes in individuals who carry personal vulnerabilities, or diatheses (Monroe and Simons, 1991; Heim and Nemeroff, 1999). A substantial body of research supports this type of model and, in particular, highlights interactions between personal characteristics and interpersonal stressors on maladaptation in at-risk youth and young adults (e.g., Hammen et al., 1995; Shahar et al., 2004; Flynn and Rudolph, 2007, 2010; Agoston and Rudolph, 2011).

A contemporary refinement of vulnerability models posits that individual variation in contextual sensitivity can take the form of plasticity, as reflected in reactivity to both disadvantageous and beneficial contexts. Specifically, differential susceptibility theory (Belsky et al., 2007, 2009; Belsky and Pluess, 2009) proposes that personal and environmental characteristics interact to predict heightened risk in adverse milieus *and* enhanced adaptation in supportive settings (for a similar model of biological sensitivity to context, see Boyce and Ellis, 2005). For instance, children with a difficult temperament, or a propensity toward negative emotionality and reactivity, demonstrate more responsiveness to certain facets of caregiving, such as supportiveness (Stright et al., 2008), sensitivity (Bradley and Corwyn, 2008; Pluess and Belsky, 2010; Roisman et al., 2012), enriching engagement (Bradley and Corwyn, 2008), and characteristics of non-maternal child care (e.g., type, quantity; Pluess and Belsky, 2010). This research reveals that children with a difficult temperament experience more adverse developmental outcomes in the presence of low quality caregiving but more beneficial developmental outcomes in the presence of high quality caregiving (Bradley and Corwyn, 2008; Stright et al., 2008; Pluess and Belsky, 2010). Notably, support for this model thus far has been obtained during infancy and childhood. Accordingly, the first goal of this study was to examine differential susceptibility interactions between youths' emotional reactivity and attributes of the caregiving environment during early adolescence. Further, given the interpersonal context of caregiving, we investigated the specificity of moderation to youths' emotional reactivity to interpersonal versus non-interpersonal stress.

### Maternal psychopathology and youth cognitive processing

Although a large body of research highlights the negative impact of maternal psychopathology, particularly depression, on the emotional backdrop of the caregiving environment, maternal psychopathology has yet to be examined within a differential susceptibility framework. Observational studies demonstrate that depressed mothers display more sadness, anger, and disengagement (Hops et al., 1987; Field et al., 1990; Pelaez et al., 2008; for a meta-analysis, see Lovejoy et al., 2000) and less positive affect (Field et al., 1990; Feng et al., 2007) during interactions with offspring. In addition, depressed mothers demonstrate more intrusive interaction styles with offspring (Field et al., 2006), express greater criticism about their children (Goodman et al., 1994; Frye and Garber, 2005), and evince less empathic understanding while observing children's challenging experiences (Coyne et al., 2007). Finally, mothers with a history of depression are more likely to react adversely (i.e., magnify, punish, neglect), and are less responsive and supportive, in response to children's expressions of negative emotions (Shaw et al., 2006; Silk et al., 2011).

Importantly, the negative emotional climate evoked by maternal depression is likely to influence youths' cognitive representations of mothers. Specifically, cognitive representations, or schemas, of caregiving relationships are believed to emerge from prior interactions and to reflect generalized knowledge and expectancies that guide information processing (Main et al., 1985; Crittenden, 1990). Whereas a history of interactions with depressed mothers is apt to produce schemas in which negative mother-relevant information is processed more efficiently, a history of interactions with emotionally sensitive and available (non-depressed) mothers is apt to produce schemas in which positive mother-relevant information is processed more efficiently. In support of this hypothesis, research reveals that never-disordered daughters of depressed mothers selectively attend to negative emotional information while never-disordered daughters of never-disordered mothers selectively attend to positive emotional information (Joormann et al., 2007). Further, consistent with a differential susceptibility model, we anticipated that this pattern of effects would be observed in youth with high, but not low, levels of emotional reactivity to stress.

Less research has investigated how maternal anxiety influences emotional facets of the caregiving environment. Preliminary observational findings reveal that anxious mothers display less positive affect and engagement, and more criticism and withdrawal, than non-anxious mothers in interactions with their children (Whaley et al., 1999; Woodruff-Borden et al., 2002). Such similar parenting difficulties observed across depressed and anxious caregivers guide the proposal that these impairments may reflect mothers' generalized negative affect, a trait that characterizes both depression and anxiety (Lovejoy et al., 2000). However, theory also suggests that parenting styles may differ across depressed and anxious mothers due to the presence of disorder-specific cognitive styles (i.e., attentional bias to sadness in depression and attentional bias to threat in anxiety; Williams et al., 1997; Clark et al., 1999). Accordingly, we investigated whether a history of interactions with anxious mothers would produce comparable schemas to those anticipated in offspring of depressed mothers (i.e., more efficient processing of negative mother-relevant information) whereas a history of interactions with non-anxious mothers would produce comparable schemas to those anticipated in offspring of non-depressed mothers (i.e., more efficient processing of positive mother-relevant information). Again, we tested whether this pattern of effects was evident in youth with high, but not low, levels of emotional reactivity to stress.

### Study overview

The overarching goal of this study was to examine whether youths' emotional reactivity to stress determined the strength of the association between maternal psychopathology and biases in youths' cognitive processing of mother-relevant information. Moreover, we investigated whether this interactive effect was specific to (a) exposure to maternal depression versus anxiety, and (b) youths' reactivity to interpersonal versus non-interpersonal stress. Biases in the cognitive processing of mother-relevant information were assessed using a levels-of-processing (LOP) task (Rudolph et al., 1995), a behavioral paradigm designed to assess youths' cognitive schemas. Specifically, youth were unexpectedly prompted to recall a list of previously presented adjectives self-rated as descriptive of their mothers; cognitive bias was quantified as the relative recall of negative versus positive information. Maternal depression and anxiety were conceptualized along a continuum from no symptoms to diagnostic-level disorder.

## Methods

### Participants

Participants were a subset of a larger sample of 4th–8th graders and their primary female caregivers (87.5% biological mothers; 2.1% stepmothers; 2.1% adoptive mothers; 8.3% other) involved in a longitudinal study examining youth development during the transition to adolescence. Youth were included based on the availability of relevant data. Specifically, the LOP task was administered to 102 of 167 youth (administration of this task was discontinued part way through the study due to time limitations). Of the 102 youth, six did not have either interpersonal or non-interpersonal emotional reactivity ratings, thereby yielding the present sample of 96 youth. Youth who had relevant data did not differ significantly from those who did not in terms of sex, χ^2^(1; *N* = 167) = 1.16, *ns*, age, *t*_(165)_ = 0.69, *ns*, ethnicity [white vs. minority; χ^2^(1; *N* = 167) = 0.43, *ns*], or income *t*_(160)_ = 0.72, *ns*. Participants ranged in age from 9 to 14 years (50 boys, 46 girls; M age = 12.36, SD = 1.05) and were somewhat diverse in ethnicity (76.0% White, 13.5% African-American, 10.5% other). Families represented a range of socioeconomic backgrounds (total annual family income was below $30,000 for 13% of the sample and above $75,000 for 18% of the sample).

### Procedures

Youth and their primary female caregivers participated in a laboratory assessment. Written consent was provided by caregivers and written assent was provided by youth. Youth completed a behavioral task assessing cognitive processing of mother-referent information. Trained graduate students, advanced undergraduate students, and a post BA-level research assistant administered the Youth Life Stress Interview (Rudolph and Flynn, 2007) to youth and caregivers. A clinical psychology faculty member and post-doctoral student, several psychology graduate students, and a post BA-level research assistant completed a semi-structured diagnostic interview assessing maternal depression and anxiety during the previous year. Both the life stress and diagnostic interviews include a set of standardized probes; supplemental questions are flexibly generated based on previous responses from individual participants to elicit more detailed information necessary for coding. Importantly, this interview method allows for the disqualification of inaccurate endorsements (i.e., elimination of false positives) and the development of additional queries when inaccurate declines are suspected (i.e., protection against false negatives), and maximizes the amount of relevant information obtained about the nature and occurrence of stressors and symptoms. Different interviewers administered the life stress and diagnostic interviews to prevent biases and preserve the objectivity of assigned ratings (for instance, prior knowledge of life stress may influence interpretations of symptoms of psychopathology, or prior knowledge of symptoms may influence interpretations of life stress). Caregivers received a monetary compensation and youth were given a gift certificate for their participation.

### Measures

#### Cognitive bias

Youth completed a LOP task (Rudolph et al., 1995) to assess cognitive processing of mother-referent information. This task activated schemas about the mother by presenting youth with a series of interpersonally descriptive adjectives, half of which were positive (e.g., loving, fun, kind) and half of which were negative (e.g., mean, strict, unfair). Following the oral and written presentation of each word, youth were asked one of two randomized questions: (1) Does this word describe your mother? (i.e., mother-referent); or (2) Is this word in capital letters? (i.e., structural). After all adjectives were administered, youth were unexpectedly prompted to recall as many words as possible. Cognitive bias is reflected in enhanced recall of positively versus negatively valenced information.

The word list included 44 adjectives that reflected four categories of 11 words each: positive mother-referent, negative mother-referent, positive structural, and negative structural. To prevent bias due to primacy and recency effects on memory, the two first and last words, each of which represented one of the four categories, were excluded from analyses. Because of our interest in cognitive processing of mother-referent information, the present analyses focused on words encoded under the mother-referent probe and rated “yes” by youth (i.e., mother-referent words). Two scores were calculated: proportion of positive mother-referent words recalled (i.e., the number of recalled yes-rated positive mother-referent words divided by the total number of yes-rated positive mother-referent words) and proportion of negative mother-referent words recalled (i.e., the number of recalled yes-rated negative mother-referent words divided by the total number of yes-rated negative mother-referent words). Consistent with previous research (Rudolph et al., 1995), relative recall of negative versus positive mother-referent words was computed. Specifically, the proportion of yes-rated negative mother-referent words recalled was subtracted from the proportion of yes-rated positive mother-referent words recalled. Thus, higher scores reflect a more positive cognitive bias toward mother-referent information and lower scores reflect a more negative cognitive bias toward mother-referent information.

Converging lines of evidence support the validity of the LOP task. Specifically, children demonstrate greater recall of self-referent than structural or semantic words (Hammen and Zupan, 1984) and the content of children's self-schemas correlates with relevant constructs such as depressive symptoms (Hammen and Zupan, 1984; Zupan et al., 1987) and low self-esteem (Hammen and Zupan, 1984). Moreover, mother-referent LOP scores are associated with other assessments of cognitive schemas of mothers (Rudolph et al., 1995).

#### Maternal psychopathology

Interviewers administered the nonpatient version of the Structured Clinical Interview for DSM (SCID IV-NP; First et al., 1996) to assess caregivers' symptoms of depression and anxiety during the previous year. In consultation with a clinical psychology faculty member or post-doctoral student, ratings were assigned according to DSM-IV-TR (American Psychiatric Association, 2000) criteria on a 5-point scale: 0 = No symptoms, 1 = Mild symptoms, 2 = Moderate symptoms, 3 = Diagnosis with mild to moderate impairment, and 4 = Diagnosis with severe impairment. Ratings were assigned based on the number, severity, frequency, duration, and resulting impairment associated with symptoms of each type of depressive disorder (i.e., major depression, dysthymia, bipolar disorder, and depressive disorder not otherwise specified) and anxiety disorder (i.e., generalized anxiety disorder, panic disorder, agoraphobia, social phobia, post-traumatic stress disorder, obsessive-compulsive disorder, specific phobias, anxiety disorder not otherwise specified). Consistent with prior research (Davila et al., 1995; Rudolph et al., 2000, 2009; Hammen et al., 2004; Flynn and Rudolph, 2011), these ratings reflect multiple indicators and include both diagnosable episodes and subthreshold symptoms of depression and anxiety. Ratings were summed across episodes and within major categories of psychopathology to create separate continuous symptom summary scores for depression and anxiety. Thus, higher scores indicate a greater number or severity of symptoms within a single diagnostic category and/or the presence of symptoms from multiple diagnostic categories. This continuous scoring system coheres with findings from taxometric studies demonstrating that depression (Haslam, 2003; Fergusson et al., 2005; Hankin et al., 2005) and anxiety (Ruscio et al., 2001; Haslam, 2003; Kollman et al., 2006) are dimensional, as opposed to categorical, disorders.

Validity of the depression summary scores was established through a significant correlation with the anhedonic depression subscale of the Mood and Anxiety Symptom Questionnaire (MASQ; Clark and Watson, 1991; *r* = 0.31, *p* < 0.01). Validity of the anxiety summary scores was established through a significant correlation with the general distress anxiety subscale of the MASQ (*r* = 0.24, *p* < 0.05). These correlations are likely moderate given that the MASQ scores reflected only recent symptoms whereas the SCID ratings reflected past-year symptoms. Based on independent coding of audiotapes of 42 interviews, strong inter-rater reliability (one-way random-effects intraclass correlation coefficient [ICC]) was found for the depression ratings (ICC = 0.98) and the anxiety ratings (ICC = 0.97). Of the 96 caregivers, 16% met diagnostic criteria for a depressive disorder (a rating of three or four for at least one episode) during the prior year; an additional 15% experienced subclinical depressive symptoms (i.e., a rating of one or two for at least one episode). Thirty-eight percent met diagnostic criteria for an anxiety disorder during the prior year; an additional 28% experienced subclinical anxiety symptoms.

#### Emotional reactivity

Interviewers administered the Youth Life Stress Interview (Rudolph and Flynn, 2007), an adaptation of the Child Episodic Life Stress Interview (Rudolph and Hammen, 1999; Rudolph et al., 2000) separately to youth and caregivers. This semi-structured interview assesses the incidence and intensity of episodic stressors experienced by youth during the prior year using the contextual threat method (Brown and Harris, 1978). Following a general query probing exposure to any type of stressful experiences, a sequence of standardized questions was asked to determine the occurrence of episodic stressors in a variety of life domains (e.g., family, peer, and romantic relationships; academics; health). Events were categorized by a team of coders as interpersonal (events that involved a significant interaction between the youth and another person or that directly affected the relationship between the youth and another person) or non-interpersonal (all other events) (Cohen's *k* = 0.92 for classification of event content). Immediately following youths' report of each event, they provided ratings on a 5-point scale (1 = *Not at All* to 5 = *Very Much*) of the extent to which they felt sad, scared/worried/nervous, angry/mad, and guilty following the event. Average emotional reactivity scores were calculated separately for interpersonal and non-interpersonal events by taking the mean of all four emotion ratings across the relevant events.

## Results

### Preliminary correlational analyses

All analyses were conducted using SPSS Statistics Version 19 software. Table 1 presents descriptive information and correlations among the variables. Significant positive correlations were found between interpersonal and non-interpersonal emotional reactivity, and between maternal depression and anxiety. A marginally significant positive association was found between maternal depression and a negative cognitive bias.

**Table 1:** **Descriptive information and intercorrelations among the variables**.

**Measure**	***M***	**(SD)**	**1**	**2**	**3**	**4**	**5**
Cognitive bias	0.07	(0.32)	–				
Interpersonal emotional reactivity	2.10	(0.73)	−0.03	–			
Non-interpersonal emotional reactivity	2.02	(0.72)	−0.02	0.48^**^	–		
Maternal depression	0.89	(1.54)	−0.18^*^	0.03	0.02	–	
Maternal anxiety	3.21	(3.49)	−0.05	0.07	0.10	0.30^**^	–

* *p* < 0.10;

** *p* < 0.01.

### Examination of moderation

Two hierarchical multiple regression analyses were conducted to examine whether emotional reactivity moderated the association between maternal psychopathology and cognitive bias in the processing of mother-referent information. In each analysis, the mean-centered main effects of maternal psychopathology and emotional reactivity were entered in the first step, and the Maternal Psychopathology × Emotional Reactivity interactions were entered in the second step. Separate regressions tested the specificity of the interactive effects to interpersonal versus non-interpersonal emotional reactivity. Significant interactions were interpreted by solving the unstandardized regression equations to predict cognitive bias from maternal psychopathology at high (one standard deviation above the mean), medium (mean), and low (one standard deviation below the mean) levels of emotional reactivity (Aiken and West, 1991).

**Figure 1:**
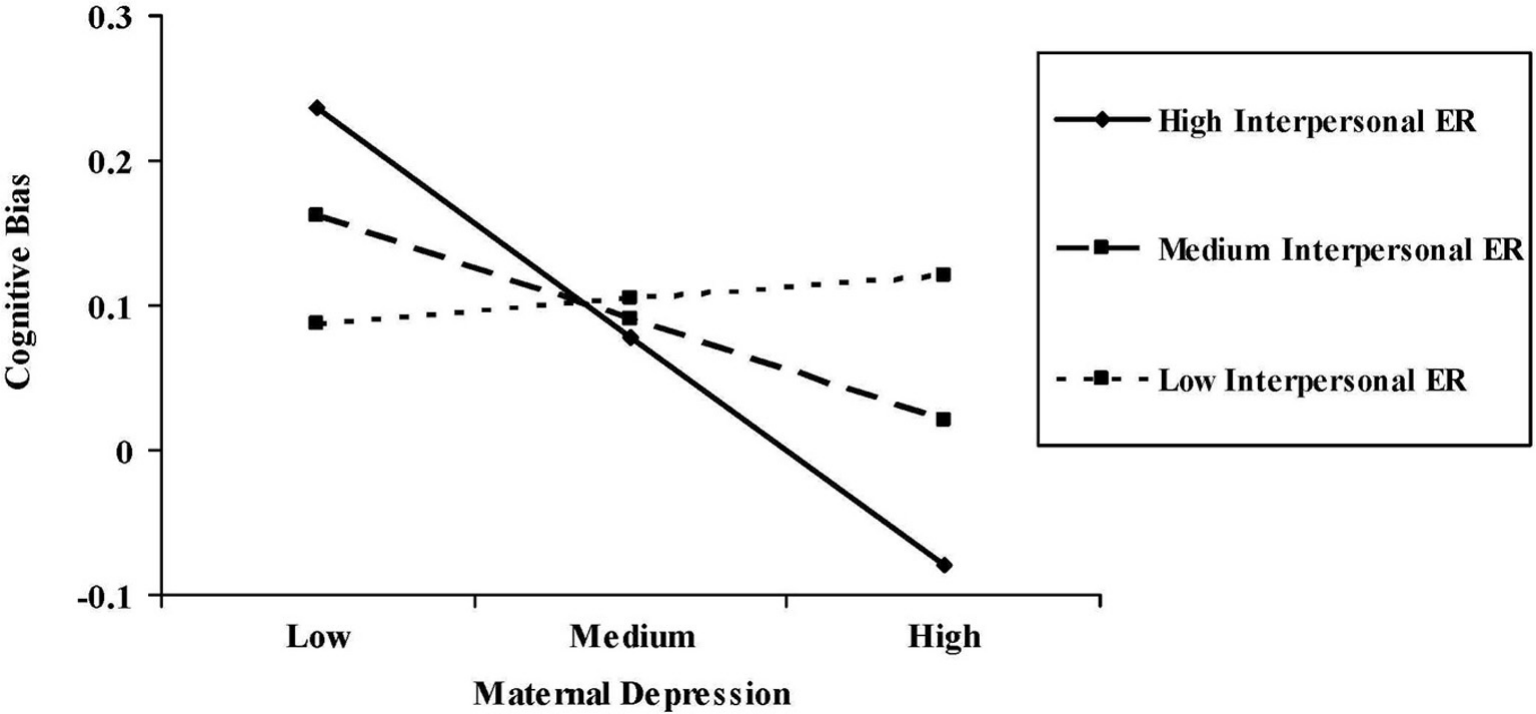
**Interaction between maternal depression and interpersonal emotional reactivity predicting youths' cognitive bias during the processing of mother-relevant information.** Negative scores on the *y*-axis indicate greater negative cognitive bias; positive scores on the *y*-axis indicate greater positive cognitive bias.

Results from the first regression revealed a marginally significant negative main effect of maternal depression, and nonsignificant main effects of maternal anxiety and interpersonal emotional reactivity. Examination of the interaction terms revealed that interpersonal emotional reactivity moderated the effect of maternal depression, but not maternal anxiety, on cognitive bias (Table 2, Regression 1). Decomposition of this interaction revealed that maternal depression was significantly negatively associated with cognitive bias (higher scores reflect more positivity) in youth exhibiting high (β = −0.52, *t*_(82) = −3.21,_ *p* < 0.01) and average (β = −0.23, *t*_(82)_ = −2.05, *p* < 0.05), but not low (β = 0.06, *t*_(82)_ = 0.39, *ns*), levels of interpersonal emotional reactivity (Figure 1). At high levels of maternal depression, youth with high interpersonal emotional reactivity showed negative cognitive biases 0.65 SDs stronger than youth with low interpersonal emotional reactivity. At low levels of maternal depression, youth with high interpersonal emotional reactivity showed positive cognitive biases 0.48 SDs stronger than youth with low interpersonal emotional reactivity.

**Table 2:** **Predicting cognitive bias in processing of mother-relevant information**.

	**Predictors**	***B***	***t***
**REGRESSION 1**			
Step 1	Maternal depression	−0.22	−1.91^*^
	Maternal anxiety	0.04	0.33
	Interpersonal emotional reactivity (ER)	−0.03	−0.24
Step 2	Maternal depression × Interpersonal ER	−0.26	−2.19^**^
	Maternal anxiety × Interpersonal ER	−0.02	−0.15
**REGRESSION 2**			
Step 1	Maternal depression	−0.16	−1.39
	Maternal anxiety	−0.01	−0.05
	Non-interpersonal emotional reactivity (ER)	−0.02	−0.17
Step 2	Maternal depression × Non-interpersonal ER	0.19	1.67
	Maternal anxiety × Non-interpersonal ER	−0.09	−0.79

* *p* < 0.10;

** *p* < 0.05.

Results from the second regression revealed nonsignificant main effects of maternal depression, maternal anxiety, and non-interpersonal emotional reactivity on youths' cognitive bias. Further, non-interpersonal emotional reactivity did not moderate the effect of maternal depression or maternal anxiety on cognitive bias.

## Discussion

Findings from this research support a novel integrated emotion-cognition differential susceptibility model in which youths' sensitivity to context predicted a more adverse developmental outcome in the presence of a low quality caregiving environment but a more beneficial developmental outcome in the presence of a high quality caregiving environment during early adolescence. Specifically, whereas exposure to maternal depression predicted a stronger *negative* bias during cognitive processing of mother-relevant information in youth exhibiting high and average levels of interpersonal emotional reactivity, youth with heightened interpersonal emotional reactivity showed a stronger *positive* cognitive bias at low levels of maternal depression. In contrast, maternal depression did not predict cognitive bias in youth exhibiting low levels of interpersonal emotional reactivity. Notably, this interactive effect was specific to interpersonal (but not non-interpersonal) emotional reactivity and to maternal depression (but not anxiety).

Emotional reactivity as assessed in this research reflects youths' negative emotional reactions (i.e., sadness, anxiety, anger, guilt) to a comprehensive array of naturally occurring stressors experienced during the prior year. Importantly, youth who display more emotional reactivity in response to stress signal their distress to others. In the context of emotionally sensitive and available (non-depressed) mothers, the expression of heightened emotional distress likely prompts mothers to engage youth in adaptive emotion regulatory and coping processes. Consequently, offspring of mothers with low levels of depression might attend to, encode, or recall interactions with mothers in a more positive manner. Conversely, depressed mothers may experience difficulty identifying and implementing effective strategies to help highly emotionally reactive youth relieve distress. In turn, offspring of depressed mothers might attend to, encode, or recall interactions with mothers in a more negative manner.

The specificity of these results to maternal depression, but not anxiety, is consistent with intergenerational models of depression transmission. Specifically, beyond the direct expression of depressive symptoms, such as anhedonia, irritability, and fatigue, depression is uniquely accompanied by a cognitive style characterized by maladaptive self-perceptions and internal, stable, and global attributions about negative events (Beck, 1967; Abramson et al., 1989). Research suggests that depressed mothers transmit this excessive emotional and cognitive negativity to offspring through their parenting behaviors (for a review, see Goodman and Gotlib, 1999), thereby transferring markers of depression risk, including attentional biases toward negative emotional information (Joormann et al., 2007), to youth. In fact, whereas non-depressed youth show a positive cognitive bias when recalling mother-referent adjectives on the LOP, youth with elevated depressive symptoms do not (Rudolph et al., 1997). Moreover, when jointly examined, depression (but not anxiety) predicts greater relative negativity during children's cognitive processing of mother-relevant information (Rudolph et al., 1997). Together with the present results, these findings suggest that negatively biased information processing may reflect one mechanism contributing to the intergenerational transmission of depression.

Additionally, maternal depression interacted with youths' emotional reactivity to interpersonal, but not non-interpersonal, stress to predict biases in youths' cognitive processing. This finding coheres with theory (e.g., Coyne, 1976; Hammen, 1991) and research (e.g., Rudolph et al., 2009; Flynn and Rudolph, 2011) emphasizing the specific role of interpersonal stress in depression onset and continuity. Heightened emotional reactivity likely perpetuates dysregulated interpersonal stress responses and interferes with the formulation of adaptive coping, particularly when mothers are unable to redirect youth toward efficacious reactions. Difficulty resolving interpersonal stress may cause youth to negatively process information about relationships, perhaps intensifying interpersonal discord and generating risk for depression. In contrast, emotionally sensitive mothers may recognize youths' maladaptive reactivity to interpersonal stress and implement strategies to facilitate constructive coping responses. Accordingly, youth who successfully negotiate interpersonal disturbances may positively process information about relationships and experience protection against depression.

## Strengths, limitations, and conclusions

Overall, this research represents a novel investigation of emotion-cognition linkages framed within a differential susceptibility model, and includes several methodological strengths. First, use of a behavioral paradigm to index cognitive processing eliminated distortion due to response biases such as social desirability, which may occur when informants select responses that will be viewed favorably by others (e.g., the endorsement of positive but not negative maternal attributes). Second, emotional reactivity was assessed in response to naturally occurring events, thereby minimizing confounds associated with estimating reactions to hypothetical stressors. Finally, the administration of semi-structured diagnostic interviews provided a comprehensive and refined assessment of maternal psychopathology.

In spite of these strengths, several limitations are worth noting. First, maternal psychopathology served as a proxy for the emotional quality of caregiving experiences; it would be helpful in future research to assess specific parenting behaviors during mother-child interactions (e.g., maternal sensitivity) that may shape youths' cognitive processing. Second, the study included a relatively small sample of youth, in which only a subset of caregivers experienced diagnoses or subclinical symptoms of psychopathology. Thus, future research will need to replicate these findings in a large, ethnically diverse sample of youth as well as in samples of caregivers with diagnostic levels of psychopathology. Third, our emotional reactivity index reflected the experience of negative emotionality in response to stress. Although this index is consistent with the construct of difficult temperament, which is the focus of theory and research on differential susceptibility, it is unclear whether the cognitive benefits accrued to youth with high emotional reactivity resulted from non-depressed mothers' ability to react in an emotionally supportive manner when youth are stressed or whether the same youth also displayed heightened positive emotionality in response to support, thereby resulting in positive cognitive biases. Finally, this study specifically examined cognitive biases during the processing of mother-referent information, and it remains to be determined whether results generalize to youths' cognitive processing of other relationships (e.g., peers, siblings) or non-interpersonal domains (e.g., academics, health).

In sum, these findings illuminate one personal characteristic of youth that shapes emotion-cognition linkages during early adolescence, and reveal trade-offs of emotional reactivity for cognitive processing such that both enhancing *and* impairing effects emerge as a function of socialization environment. That is, in the context of maternal depression, youths' heightened emotional arousal and distress may impair cognition by generating a perseverative focus on negative features of the environment, including information about emotionally insensitive or unavailable caregivers. In contrast, in parenting contexts characterized by low maternal depression (and, perhaps, accompanying warmth and sensitivity), youths' emotional reactivity may enhance cognition by allowing youth to interpret caregiving interactions in a positive light. Given that negative cognitive biases represent a risk factor for depression, these findings implicate youths' emotional reactivity and maternal depression as joint targets of intervention and prevention endeavors. Overall, this research emphasizes the importance of considering integrative, developmentally sensitive perspectives of the complex interplay between emotion and cognition, which may involve mutually enhancing or impairing associations, particularly as emotion-cognition linkages pertain to the onset and maintenance of psychopathology across the lifespan.

### Conflict of interest statement

The authors declare that the research was conducted in the absence of any commercial or financial relationships that could be construed as a potential conflict of interest.
